# Novel high protein-energy balls formulated with date paste enriched with Samh seeds powder and/or different milk protein origins: effect on protein digestibility *in vitro* and glycemic response in young adults

**DOI:** 10.3389/fnut.2025.1538441

**Published:** 2025-03-26

**Authors:** Hathami Alsuhebani, Sally S. Sakr, Hany Elkashef, Reham M. Algheshairy, Hani A. Alfheeaid, Metab Algeffari, Hend F. Alharbi

**Affiliations:** ^1^Department of Food Science and Human Nutrition, College of Agriculture and Food, Qassim University, Buraydah, Saudi Arabia; ^2^Dairy Foods Department, Faculty of Agriculture, Cairo University, Giza, Egypt; ^3^Department of Family and Community Medicine, College of Medicine, Qassim University, Buraydah, Saudi Arabia; ^4^Abdullah Al-Othaim Diabetes Center, Medical City, Qassim University, Buraydah, Saudi Arabia

**Keywords:** energy balls, Samh seeds, camel milk, protein, digestibility, glycemic response, antioxidants, food intake

## Abstract

**Objectives:**

The rising demand for convenient and nutritious food options, especially among young adults with fast-paced lifestyles, highlights the need for quick energy and protein sources during physical activities and breakfast. Consequently, aimed to formulate and evaluate the nutritional, functional, and glycemic properties of high-protein energy balls using Sukkari date paste a variety of the date palm (*Phoenix dactylifera* L.) paste, Samh seed (*Mesembryanthemum forsskalei* Hochst) powder, whey protein concentrate, and camel milk powder.

**Methods:**

The nutritional value of the formulated balls was evaluated by assessing their chemical composition, dietary fibers, amino acids (AAs), and fatty acids (FAs). Additionally, antioxidant properties were determined using the DPPH method and reducing power assays. *In vitro* protein digestibility was also measured. Furthermore, the *in vitro* glycemic index and glycemic load, as well as the human glycemic response for various samples, were examined.

**Results:**

Samples containing combinations of date paste, Samh seeds, and either camel milk powder (DSC) or whey protein concentrate (DSW) demonstrated high nutritional value, with significant caloric content measured at 352.76 ± 0.125 Kcal/100 g for the first combination and 328.76 ± 0.242 Kcal/100 g for the second. These samples also showed significant (*p* < 0.05) DPPH radical scavenging activity, with values of 63.78 ± 2.43 μg of ascorbic acid equivalent/g for the date paste and Samh seeds with camel milk powder (DSC) and 59.87 ± 2.61 μg of ascorbic acid equivalent/g for those with whey protein (DSW). Furthermore, the presence of a variety of essential amino acids and fatty acids in DSC and DSW was higher than in the rest of the samples (DS, DW and DC), which is under the current study. Sensory evaluations indicated that all samples were highly accepted. The *in vitro* study revealed that the degree of protein digestibility was higher in samples that contained both Samh seeds powder and whey protein concentrate or camel milk powder than in the sample that contained Samh alone. Also, all samples exhibited low *in vitro* glycemic index (<55) and glycemic load (<10). Moreover, the human glycemic response evaluation showed that blood glucose levels gradually declined after 30 min, returning to pre-meal levels by 120 min, indicating no post-meal hyperglycemia, resulting in a normal glycemic response in healthy young adults.

**Conclusions:**

Combining Samh seed powder with dairy proteins to create protein-energy balls using Sukkari date paste results in nutritious snacks that are rich in amino acids, fatty acids, dietary fibers, and antioxidant compounds. These snacks also have a low glycemic response and high protein digestibility *in vitro*. Therefore, high-protein energy balls made from date paste enriched with Samh seed powder, along with either camel milk or whey protein powders, are recommended as a protein and energy source for healthy young adults who do not experience post-meal hyperglycemia.

## 1 Introduction

The demand for nutraceuticals, functional components, or diets is rising as a result of consumers' unhealthy eating habits, malnutrition or overnutrition, and the negative consequences of synthetic medications. Moreover, the considerable alterations in the dietary behaviors of consumers due to a quick civilization, and modernistic lifestyle influenced the forms of meals in the global market. Hence, bars, snacks, balls, or powder forms were developed as substitutes for wholesome meals (1). Recently, the preparation of novel types of these forms using functional or healthy ingredients has been given significant attention, taking into consideration consumers' acceptability and appropriateness of these meals as ready-to-eat (2). Also, different forms of energy snacks (bars, balls, bites) are popular food products that are often consumed as nutritious and convenient meals. These snacks contain various ingredients to cater to different ready-to-carry dietary preferences and are especially used to boost athletic performance and meet daily energy requirements for young adults. Research indicates that energy snacks can be a balanced and nutritious option when made with carefully selected ingredients. This not only enhances consumer acceptance but also supports trends toward food sustainability (3–5). High-protein balls or bars are one of the rapid-augmenting meals on the global market. These snack forms contain more than 20 g of protein/serving and a high fiber content, but they have a low content of carbohydrates and sodium (6). Athletes and health-conscious consumers have recourse to high-protein bars or balls as well. In fact, these snacks may be a safe and efficient approach to weight loss in obesity and overweight cases; hence, they may be crucial for the protection of dyslipidemia, diabetes, and other metabolic disorders (7).

The preparation of high protein-energy balls should comprise premium raw ingredients with high bioactive components. The formulation of energy snacks incorporates diverse ingredients to optimize nutritional value and functional benefits. At the core of these formulations are dried fruits, nuts, and seeds, with dates serving as the predominant dried fruit component, comprising 55% to 90% of the formulation. Their inclusion is largely due to their impressive nutritional profile, particularly their antioxidant activity and anti-inflammatory properties (3, 5). To enhance the nutritional density of energy bars, almonds and various oilseeds are frequently integrated, providing substantial protein and healthy fats. This not only increases the energy density of the bars but also amplifies their health benefits. Furthermore, the incorporation of whole grains such as oats, along with legume flours derived from beans, is strategically employed to boost fiber and protein content. This combination enhances the overall macronutrient profile, facilitating sustained energy release (8). In addition to these core ingredients, functional additives are critical for bolstering the bars' overall efficacy. Dairy protein sources, including skim milk powder and various protein concentrates, are utilized to significantly elevate protein density, thereby catering to the dietary needs of athletes and individuals seeking to increase protein intake. Additionally, plant extracts—derived from various plant parts such as leaves and seeds—are incorporated to enrich the phytochemical content, particularly phenolics and flavonoids. This enhancement not only improves the antioxidant potential of the snacks but also contributes to their overall health-promoting characteristics (4, 9).

Sustainability and sensory acceptance present significant challenges, especially with the growing interest in creating energy snacks that utilize sustainable and environmentally friendly ingredients. This includes using by-products to minimize nutrient waste while supporting a productive economy (4, 5). Additionally, sensory attributes such as taste and texture play a crucial role in consumer acceptance of snacks. These properties can be enhanced through carefully selecting ingredients and efficient processing methods (8, 9). In this regard, Camel milk, dates, whey protein powders, and Samh seeds represent innovative and sustainable food sources with significant nutritional value (3, 10, 11). Camel milk is rich in bioactive compounds and essential nutrients, and its production is particularly well-suited to arid and semi-arid environments, aligning with the adaptive capabilities of camels to thrive under such conditions which made them attractive for consumers not only in the Gulf but in European markets as well (12). Dates, a staple in Middle Eastern diets (3), and Samh, an underutilized seed from desert plants (11), demonstrate resilience to high temperatures and salinity, making them viable agricultural products in the face of climate change and escalating desertification. These ingredients not only provide essential nutrients but also contribute to sustainable food systems by leveraging crops that can endure increasingly harsh climatic conditions. The integration of these elements into dietary practices holds promise for enhancing food security and nutritional quality in regions prone to environmental stress. In this respect, camel milk is recognized as superior because of its unique attributes compared to other milk species. Camel milk exhibits various therapeutic effects against human diseases, including metabolic and chronic disorders. These effects are due to its high content of bioactive substances and the release of bioactive peptides from its original proteins during the passage in the gastrointestinal (13). In addition, milk proteins, particularly whey proteins, have earned prevalent publicity for their health effects in muscle building and weight administration. Whey proteins contain a high amount of essential amino acids, and they are an important source of branched-chain and sulfur-containing amino acids (14). Thus, the use of camel milk or whey proteins as functional ingredients has obtained significant attention in the field of functional food industry and sport.

Although the replacement of animal proteins with plant proteins still has the contrary point of view, a respectable development has been reported in the global market for vegetarian consumers. The Dietary Guidelines Advisory Committee 2015 recommended that empty calories in added sugars should be partially substituted with a good diversity of vegan protein (15). One of the most important sources of plant proteins is Samh seeds. This plant is widespread and grows normally in different regions of Arabic countries such as Egypt, Saudi Arabia, Qatar, and Kuwait, and it is known as forskal fig-marigold in Aljouf, Saudi Arabia (16). Awabdeh et al. (17) reported that Samh seeds are rich in protein (about 23%) and comprise 4.4% fat, 9.1% crude fiber, 6.6% ash, and 11.5% moisture. Several studies have reported that Samh seeds have diverse health effects, including enhancement of kidney functions, antioxidant properties, antimicrobial activity, improvement of gastrointestinal status, and cytotoxic ability against LoVo cancer cell lines was also noted (18, 19). In this context, the Palm date tree is one of the ancient trees globally. On fresh weight, dates are rich in sugars (38–50%) and contain 1.1% proteins, 0.1% fat, 1.0–1.4% ash, and 37–50% moisture (20). Functionally, Biglari et al. (21) found many flavonoids in dates, including apigenin, ferulic, sinapic, quercetin, and p-coumaric acids. As known, these components have various health impacts on the human body, such as a decrease in capillary weakness, activation of the immune system, ability to bind oxidants, anti-inflammatory, and from strong to moderate anticancer characteristics (20–22).

Various commercially available high-protein snacks are produced by plant mixtures, whilst just a little are evaluated on a scientific basis. Also, the manufacturers of high-protein snacks or bars meet different challenges, including the components that should have a high bioavailability and the consumer acceptability of these products and their health effects. At the same time, the need for healthy and ready-to-eat foods in the busy and fast lifestyle in different societies is increasing markedly. This eating behavior, especially among young adults, has become trendy. Thus, the need for quick energy and protein sources during sports activities and breakfast is increasing.

This study presents a novel approach to formulating and assessing high-protein energy balls utilizing an innovative combination of ingredients: Samh seeds, camel milk powder, date paste, and whey protein concentrate. The evaluation assessed their nutritional value, antioxidant properties, *in vitro* glycemic index, and *in-vitro* protein digestibility. Additionally, the glycemic response to these energy balls was measured in young adults.

## 2 Materials and methods

### 2.1 Materials

Samh seeds powder (92.34% total solids, 19.21% total protein, 5.51% fat, 2.81% ash, 64.82% carbohydrates, and 13.29% dietary fibers) cultivated in Al-Jouf province, Saudi Arabia (KSA) was purchased from local market. Sukkari date paste (79.09% total solids, 2% total protein, 0% fat, 1.69% ash, 75.40% carbohydrates, and 8% dietary fibers) was procured from the local market in Buraydah, Qassim region, KSA. Camel milk powder (CMP: 25% total protein, 25% fat, and 40% lactose) was imported from Camelicious company, Dubi, United Arab Emirates. Whey protein concentrate (WPC: 79.17% protein, 6.25% fat, and 40% lactose) produced by Now Foods company, USA, was brought from an online supplier. The specific chemicals for each experimental method are outlined according to their respective analysis method and were subsequently described.

### 2.2 Study design

**Figure 1:**
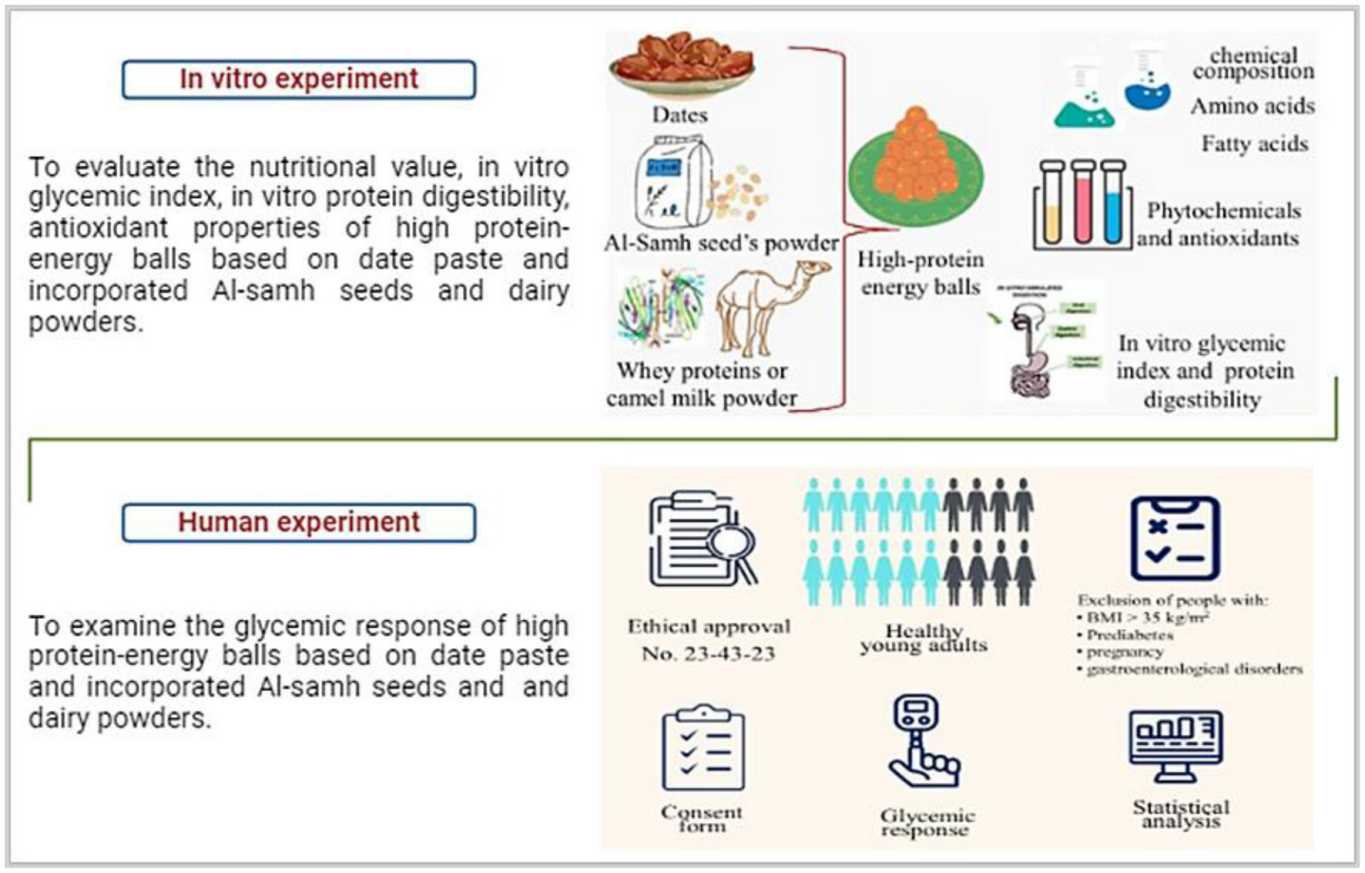
Illustration of the study design.

The current study was performed under the approval of the Committee of Research Ethics, Deanship of Scientific Research, Qassim University, Saudi Arabia (Approval No. 23-43-23). The study encompassed both *in vitro* and *in vivo* experiments. All human *in vivo* experiment participants were duly informed that their data would be utilized for scientific research, and everyone provided a signed consent form. The study design is visually depicted in Figure 1.

### 2.3 High protein-energy balls formulation

**Figure 2:**
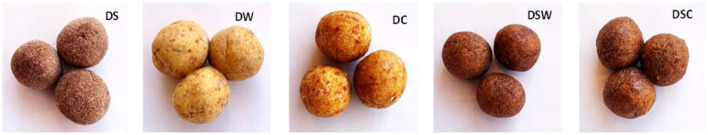
Photos of different formulated high protein-energy balls. *DS*, High protein-energy ball formulated with Sukkari date paste and Samh seed's powder; *DW*, High protein-energy ball formulated with Sukkari date paste and WPC; *DC*, High protein-energy ball formulated with Sukkari date paste and CMP; *DSW*, High protein-energy ball formulated with Sukkari date paste, Samh seed's powder and WPC; and *DSC*, High protein-energy ball formulated with Sukkari date paste, Samh seed's powder and CMP.

Samh five different energy balls (Figure 2) formulas were prepared depending on the chemical composition of each component used in the study, particularly total proteins and total carbohydrates content. The differences between high protein energy ball formulations are presented in Table 1. The quantities of each ingredient used to prepare the energy balls were calculated to yield 100 g each. The protein content in these energy snacks was designed to contribute at least 10% of the total calories. Consequently, the amount of ingredients, excluding date paste, was calculated to provide 8% of the total protein percentage. Each ingredient's contribution was then determined to ensure that more than 10% of the calories in each formula came from protein. The results were as follows: DS: 10.7%, DW: 12.3%, DC: 10.3%, DSW: 10.4%, and DC: 10.4%. Additionally, this formulation aimed to provide approximately 10% of the daily protein calorie needs for young adults (about 40 out of 400 kcal of protein per day), based on dietary reference values for nutrients reported by (23).

**Table 1:** Different high protein-energy balls formulation.

**High protein-energy ball**	**Ingredients (g/100 g)**			
	**Samh powder**	**Sukkari date paste**	**WPC**	**CMP**
DS	41	59	–	–
DW	–	90	10	–
DC	–	68	–	32
DSW	20	75	5	–
DSC	20	64	–	16

DS, High protein-energy ball formulated with Sukkari date paste and Samh seed's powder; DW, High protein-energy ball formulated with Sukkari date paste and WPC; DC, High protein-energy ball formulated with Sukkari date paste and CMP; DSW, High protein-energy ball formulated with Sukkari date paste, Samh seed's powder, and WPC; and DSC, High protein-energy ball formulated with Sukkari date paste, Samh seed's powder and CMP.

### 2.4 Analysis of prepared high protein-energy balls

#### 2.4.1 Chemical composition and nutritional value of high-protein energy balls

Chemical constituents of ingredients and samples of final products (total solids, total protein, fat, ash, crude fiber and dietary fiber) were determined according to the AOAC (24). Total carbohydrates were calculated by difference. The nutritive value was calculated based on the proportion of ingredients used in the formulation of the high protein-energy balls by applying the following equation:


$$\begin{array}{c} \text{Energy }\left(\text{k}.\text{cal}\right)\;=\left[\text{Protein }\left(\text{g}\right)\;\times \;4\right]\;+\;\left[\text{Carbohydrate }\left(\text{g}\right)\;\times \;4\right]\\ \;+\left[\text{Fat}\left(\text{g}\right)\;\times \;9\right]. \end{array}$$

The percentage of the daily value (%DV) for each formulation of energy balls was estimated based on the contribution of its components to a 2,000-calorie daily diet per 100 g of each ball's formula.

#### 2.4.2 Fatty acids profile analysis

The variance in fatty acids (FAs) content and their relative distribution in different prepared high-protein energy balls formulations were determined by Gas Chromatography (GC). The GC model 7890B from Agilent Technologies was equipped with a flame ionization detector at Central Laboratories Network, National Research Centre, Giza, Egypt. Separation was achieved using a Zebron ZB-FAME column (60 m × 0.25 mm internal diameter × 0.25 μm film thickness). Analysis was carried out using hydrogen as the carrier gas at a flow rate of 1.8 mL/min at a split-1:50 mode, injection volume of 1 μL and the following temperature program: 100°C for 3 min; rising at 2.5°C/min to 240°C and held for 10 min. The injector and flame ionization detector were held at 250°C and 285°C, respectively.

#### 2.4.3 Amino acids profile analysis

The types of different amino acids (AAs) and their relative distribution in prepared high-protein energy balls were evaluated. For extraction, 0.2 g of each sample was mixed with 5 mL H_2_O and 5 mL of HCl (6 M) and then heated at 100°C for 24 h before filtration. Thereafter, 1 mL of the filtrate was dried and resuspended in HCl (0.1 M) and injected into the HPLC. The HPLC analysis was carried out using an Agilent 1260 series. The separation was carried out using the Eclipse Plus C18 column (4.6 mm × 250 mm i.d., 5 μm) maintained at 40°C. The mobile phase consisted of buffer (sodium phosphate dibasic and sodium borate), pH 8.2 (A) and ACN: MeOH: H_2_O 45:45:10 (B) at a flow rate of 1.5 ml/min. The mobile phase was programmed consecutively in a linear gradient. The Diode Array Detector (DAD) was monitored at 338 nm (Bandwidth 10 nm). The fluorescence detector was adjusted from 0 to 27 min at 340/450 nm (Excitation/Emission) and from 27 to 35 min at 266/306 (Excitation/Emission).

#### 2.4.4 Phenolic and flavonoid compounds

The food safety laboratory at the dairy department of the Faculty of Agriculture, Cairo University in Cairo, Egypt referenced the Agilent application note, publication number 5991-3801EN, 2014. The analysis involved the application of the Agilent 1260 Infinity HPLC series (Agilent, USA), featuring a quaternary pump and a Kinetex^®^ 17μm EVO C18 50 mm × 2.1 mm column (Phenomenex, USA), operated at a temperature of 30°C. The separation process utilized a ternary linear elution gradient with the following solvents: (A) HPLC grade water with 0.1% H3PO4 (v/v), (B) acetonitrile with 0.1% H_3_PO_4_ (v/v), and (C) methanol. The flow rate was calibrated at 0.2 mL/min, and the injected volume was 20 μL. Detection was performed using a variable wavelength detector (VWD) at 280 nm.

#### 2.4.5 Methanolic extract preparation

A methanolic extract was prepared in accordance with the method outlined by Saleh et al. (25). Subsequently, it was meticulously collected and then frozen at −25°C to facilitate the subsequent analysis of its antioxidant properties.

#### 2.4.6 DPPH radical scavenging activity

The DPPH radical scavenging activity of the samples was determined in accordance with the method outlined by Abd El-Fattah et al. (26). The percentage of radical scavenging activity was then calculated according to the following formula:


$$\begin{array}{c} \text{DPPH radical scavenging activity }\left(\text{\%}\right)\;=\left(\text{A}0\;-\text{ As}\right)/\text{A}0\;\times \;100. \end{array}$$

Where A0 is the absorbance at 517 nm of blank, As is the absorbance at 517 nm of extract, also, the calculation of the μg ascorbic acid equivalent per gram of the sample utilized a standard curve equation based on ascorbic acid.

#### 2.4.7 Reducing power

The reducing power assay, as referenced by Abd El-Fattah et al. (26), was conducted using the prepared methanolic extract. A standard curve of ascorbic acid (0–500 μg/mL) was generated, and the standard curve equation was applied to calculate the amount of ascorbic acid equivalent/g of the sample.

#### 2.4.8 *In vitro* glycemic index (GI) and glycemic load (GL)

The glycemic index was determined in accordance with the methodology established by Aribas et al. (27) with certain modifications. Initially, 0.1 g of each sample was meticulously weighed and placed into separate 50 mL falcon tubes. Subsequently, 2 mL of HCl (0.05 M) solution containing pepsin (0.117 g/mL, Sigma) was introduced into the tubes, followed by a 37°C-incubation in a shaking water bath for 30 min. Post-incubation, each tube received 4 mL of sodium acetate buffer (0.5 M, pH 5.2), 1 mL of an enzyme solution comprising 0.243 g of pancreatin (Sigma), and 14.45 U (56 μL) of amyloglucosidase derived from Aspergillus Niger (260 U/mL, Sigma). The tubes were then horizontally incubated at 37°C in a shaking water bath. Thereafter, aliquots (100 μL) were extracted at 20-min intervals over a 160-min duration and combined with 1 mL of absolute ethanol. Following centrifugation at 800 × g for 10 min, the glucose content was quantified by the enzymatic colorimetric method utilizing the Biodiagnostic glucose determination kit (Biodiagnostic company, Giza, Egypt), and the absorbance was measured using a Shimadzu UV-1800 spectrophotometer at a 500 nm wavelength. The absorbance values were graphed over time to produce the hydrolysis curve. To calculate the GI, the hydrolysis index (HI) of each sample was first calculated by using the following equation:

HI = The area under the hydrolysis curve of the sample / The area under the hydrolysis curve of white bread.

Then, the *in vitro* GI was determined by using the following equation:


$$\begin{array}{c} \text{GI}=39.71\;+0.549\text{HI}. \end{array}$$

Finally, GL was calculated for each sample from the following equation:


$$\begin{array}{c} \text{GL }=\left(\text{GI }\times \text{ available carbohydrate }\left(\text{g}\right)\right)\;/\;100. \end{array}$$

#### 2.4.9 *In vitro* protein digestibility and degree of proteolysis

The method described by Ménard et al. (28) was utilized to simulate both gastric and intestinal digestion. Briefly, for simulated gastric digestion (SIGD), 10g of different prepared balls was mixed with 20 mL of simulated gastric fluid (SGF; 94 mmol NaCl, 13 mmol KCl, adjusted to pH 2 using 1 mol HCl) in a 50 mL falcon tube. Next, the pH of the mixture was readjusted to 3 using 1 mol HCl, and 1 mL porcine pepsin (3,000 U/mL) was added. Digestion was carried out with continuous shaking in a water bath maintained at 37°C for 1 h, and the reaction was stopped by immersing tubes in an ice water bath. For intestinal digestion, 20 mL was mixed with 18 mL simulated intestinal fluid (SIF mixture containing: 10 mmol KCl, 85 mmol NaHCO_3_, 164 mmol NaCl, 3 mmol CaCl_2_) and 0.5 mL of pancreatin solution (10 mg of ≥3 × USP pancreatin/10 mL SIF without CaCl_2_). The pH was adjusted to 7, and the tubes were homogenized; then, digestion was carried out under continuous shaking in a water bath maintained at 37°C for 1 h. Heat shock treatment was used to stop intestinal digestion by immersing tubes in an ice-water bath. The free amino acid content of samples before and after *in vitro* digestion were measured using the O-phthaldialdehyde (OPA) method and they expressed as μmol L-leucine equivalent using a plotting of L-leucine standard curve ranged from 0 to 150 μmol/mL. The degree of hydrolysis was calculated as the amount of free amino acids (AN) released after digestion according to Mudgil et al. (80) using the following equation:


$$\begin{array}{c} \text{AN }=\text{AN}2\;-\text{ AN}1. \end{array}$$

AN1 and AN2 are the free amino group content of the balls before and after hydrolysis, respectively.

### 2.5 Human study design

Six young, healthy adult participants (2 males and 4 females) were enrolled to assess glycemic response and their arthrometric characteristics are presented in Table 2. Following informed written consent, all volunteers underwent a comprehensive interview process. Following the criteria established by Brouns et al. (29), inclusion parameters encompassed healthy volunteers, while exclusion parameters comprised a BMI >35 kg/m^2^, prediabetes, pregnancy, gastrointestinal disorders, gastrointestinal surgery, gastroenteritis within the preceding 6 months, smoking, or the use of any medications (30). According to the FAO/WHO guidelines (31) for glycemic response studies, the GI of food should be determined by repeated tests in at least six subjects (29). Furthermore, individuals with chronic conditions (e.g., bronchial asthma or rheumatoid arthritis) or acute illnesses (e.g., upper respiratory tract or urinary tract infection) were ineligible for participation in the study. Subjects were advised to maintain their standard diet and to avoid engaging in unusually vigorous activities until the study was concluded. It was recommended that participants stay seated and avoid excessive movement during the test (32).

**Table 2:** Key baseline of the participant's anthropometric characteristics (Mean ± SD).

**Variables**	**Total (*n =* 6)**
Age (years)	23.50 ± 3.62
Weight (Kg)	72.33 ± 13.12
Height (cm)	164 ± 9.30
BMI (Kg/m^2^)	26.67 ± 3.83
Waist/Hip ratio	0.81 ± 0.09
Baseline glucose (mg/dl)	90.33 ± 10.09

#### 2.5.1 Glycemic response

In a study on glycemic response, blood glucose levels were carefully monitored over a 2-h period in healthy individuals. The process involved thoroughly washing and drying hands, followed by extracting blood from the thumb post-sterilization with alcohol swabs using the Swiss-made Contour Next One blood glucose monitoring device, the glucose oxidase method. Recent reports have suggested that capillary rather than venous blood sampling is preferred for reliable GI testing (29, 33). The first drop of expressed blood was discarded, and the subsequent drop was used for testing. Subjects were blinded to the type of the date that they consumed.

Glucose levels were measured at specific intervals at 0 (fasting), 15, 30, 45, 60, 90, and 120 min for each sample as part of the glucose level analysis (34). Participants were meticulously screened using a questionnaire, with those deemed unfit for the study being excluded. Each participant's weight and height were meticulously recorded, and their BMI was calculated as weight (kg)/height in (m^2^). Subsequently, each participant's fasting blood sugar levels were measured after an 8-h fast using 50 ml of glucose to determine blood glucose levels. Each subject had a GI test on two different days following an 8–10 h overnight fast, with at least one “washout” day between each test. The subjects were blindly administered the 50 g equivalent carbs of the various test ball types in a random order and finished within 12 min. The GI and GL were computed in accordance with Wolever et al. (33), with strict adherence to finger sterilization protocols before and after each prick. Participants were also allowed to consume 250 ml of water during the 2-h monitoring period while instructed to stay seated and avoid excessive movement (34).

#### 2.5.2 Sensory evaluation

The five formulated high-protein energy balls were sensory evaluated at the Department of Food Science and Human Nutrition, Faculty of Agriculture and Food, Qassim University, Saudi Arabia. The study included 15 participants, comprising both academics and staff, who assessed several quality attributes: (i) appearance, (ii) flavor, (iii) aroma, (iv) texture, and (v) overall acceptability. To evaluate the acceptability of the samples, a 9-point hedonic scale was employed, with scores ranging from 1 (dislike extremely) to 9 (like extremely) (35). Before their participation, all individuals provided informed consent for the sensory evaluation. The samples were assigned random three-digit codes and presented in a randomized order to ensure the integrity and unbiased nature of the results. This sensory evaluation was conducted following approval from the Committee of Research Ethics at the Deanship of Scientific Research, Qassim University, Saudi Arabia (Approval No. 23-43-23).

### 2.6 Statistical analysis

Microsoft Excel 2016 and SPSS version 23 (SPSS Inc., Chicago, IL, USA) were used to analyze the data. The data were displayed as means ± standard deviations (SD). The variables of high-energy protein balls, such as the glycemic index, protein digestibility, degree of protein hydrolysis, and antioxidants, were examined by comparing one-way ANOVA. The glycemic response was analyzed using variance comparing ANOVA because the values are continuous. The participants in the study were displayed as frequency and mean.

## 3 Results and discussion

### 3.1 Chemical composition and nutritional value

You The data of the proximate chemical composition and nutritional value of all formulation balls are listed in Table 3. The protein content in all formulation balls ranged from 9.06 to 9.71%, except formulation balls composed of date paste, Samh powder, and camel milk powder (9.201%). As expected, date-WPC balls showed a significantly greater protein content compared to other formulation balls. This is attributed to the presence of WPC containing the highest protein level (79.14%) compared to other used ingredients. Jabeen et al. (36) found that adding whey protein isolate improved the protein level and the nutritional value of protein-energy bars. Szydłowska et al. (1) used WPC to produce high-protein organic bars with high nutritional and healthy value. Also, data presented in Table 3 shows that samples containing CMP (DC and DSC) had lower protein content compared to samples containing WPC (DW and DSW). This difference can be attributed to the higher fat content in CMP, which required a reduction in protein quantity to achieve a similar caloric contribution from protein across the various energy ball formulations, maintaining approximately 10% of the total caloric content.

**Table 3:** Caloric chemical components (g/100 g) and calories (Kcal/100 g) of different high-protein energy ball formulations.

**High-protein energy balls**	**Protein**	**Fat**	**Carbohydrates**	**Kcal/100 g**	**%DV**	**Crude fibers**	**Dietary fibers**
DS	9.06 ± 0.339^c^	2.26 ± 0.173^c^	71.10 ± 0.033^a^	340.99 ± 0.209^c^	17.05 ± 0.010^c^	10.20 ± 0.001^a^	2.70 ± 0.007^a^
DW	9.71 ± 0.016^a^	0.62 ± 0.02^e^	68.30 ± 0.042^c^	317.57 ± 0.053^e^	15.88 ± 0.003^e^	7.20 ± 0.003^c^	1.80 ± 0.003^b^
DC	9.39 ± 0.035^b^	8.03 ± 0.153^a^	64.12 ± 0.050^d^	366.37 ± 1.462^a^	18.32 ± 0.073^a^	6.80 ± 0.034^c^	1.02 ± 0.013^c^
DSW	9.30 ± 0.165b^c^	1.42 ± 0.08^d^	69.70 ± 0.021^b^	328.76 ± 0.242^d^	16.44 ± 0.012^d^	8.60 ± 0.001^b^	1.66 ± 0.008^b^
DSC	9.201 ± 0.082^b^	4.55 ± 0.043^b^	67.20 ± 0.106^e^	352.88 ± 0.215^b^	17.64 ± 0.011^b^	6.40 ± 0.000^c^	1.96 ± 0.000^b^

DS, High protein-energy ball formulated with Sukkari date paste and Samh seed's powder; DW, High protein-energy ball formulated with Sukkari date paste and WPC; DC, High protein-energy ball formulated with Sukkari date paste and CMP; DSW, High protein-energy ball formulated with Sukkari date paste, Samh seed's powder, and WPC; and DSC, High protein-energy ball formulated with Sukkari date paste, Samh seed's powder, and CMP; DV, daily value. Data in the same column with various superscript letters differ significantly.

Our results demonstrated that the balls formulated of date paste and camel milk powder had a significantly higher fat percentage and a significantly lower carbohydrate content than that of other formulation balls, thus because camel milk powder contains higher fat and lower carbohydrate content than other used ingredients. However, date-based Samh powder balls recorded the significantly highest carbohydrates, crude, and dietary fibers levels and this is due to Sukkari date paste and Samh seeds powder containing the greater carbohydrates (75.40% and 64.82%), dietary fibers (8.0 and 13.29%) percentages compared to other ingredients. Furthermore, the presence of crude or dietary fibers in other formulation balls is related to the relation to the amount of date paste or Samh powder that is contributed to these formulations. Siddeeg et al. (37) found that Sukkari date contain about 78.32% carbohydrates, 3.15% crude fibers, and < 1.0% fat. Mohammed et al. (11) reported that Samh seeds had 10% crude fiber and above 50% carbohydrates.

Results regarding the values of total calorie and daily values exhibited that there were significant variations among formulation balls, and the date-camel milk powder balls had the significant highest value of total calorie (366.37 Kcal/100 g ball) and daily value (18.32%). This is because of the high content of fat in camel milk powder (25%) compared to other formulation ingredients (0.0–6.25%). In addition, Sukkari date paste is a perfect source of dietary fibers that is favorable for the digestive system. Also, camel milk powder is a good source of protein (25.0%) and contains a great level of β-casein (~65% of total milk caseins). Plenty of β-casein in camel milk plays a crucial role in camel milk easy digestibility in the human body since β-casein is less impedance to proteolytic enzymes than αs-casein (38). Moreover, the moderate diameter of fat globules of camel milk is lower (2.99 μm) than that of other milk types, and this attribute makes fat globules more exposure to lipolysis, thus becoming more easily digested (39).

### 3.2 Amino acids (AAs) profile

The total and free AAs profile of different high protein-energy balls is listed in Table 4. Our findings revealed that the addition of WPC to date-balls increased the level of AAs due to its high containing of all essential AAs (Ile, Leu, Lys, Met, Phe, Thr, and Val) and non-essential AAs including Glu, Ser, Asp, and Ala. Moreover, the ball formulations composed Samh seeds powder and WPC recorded the second formula in the total AAs content. Our results were supported by Szydłowska et al. (1), who found that the addition of WPC to high-protein bars increased the total amino acids content, particularly essential amino acids. As known, essential AAs cannot be generated by the consumer body; hence, should be provided by food. Regarding the health advantages of whey proteins, its amino acid profile is very identical to that of skeletal muscle, supplying roughly all the AAs convergent ratio to their proportions in muscles. The plenty of Leu in whey proteins is significant because of its vital role in the metabolism of protein, which has been known as a key sign in the translation inception path of muscle protein biosynthesis in the human body. In addition, Glu plays a pivotal role in the quick dividing of cells and is considered substantial through metabolic strain or sickness periods. All essential AAs should be found in each diet at adequate levels to promote the postprandial muscle protein biosynthesis process, where essential AAs are operators that have the capability to catalyze the muscle tissue anabolic pathways (40). As for Samh seeds powder was found to be rich in protein content (20–23%) and contains plenty of His, Met, Tyr, Gly, and Arg acids (16, 17).

**Table 4:** Amino acids profile (mg/100 g) of different high-protein energy ball formulations.

**Amino acid**	**DS**	**DW**	**DC**	**DSW**	**DSC**
**Essential AAs (EAAs)**					
Histidine	5.332	2.832	2.984	2.957	3.145
Isoleucine	2.115	5.211	3.312	3.591	2.606
Leucine	3.479	8.851	5.828	5.850	4.546
Lysine	4.801	8.118	6.872	5.756	5.456
Methionine	0.847	1.180	1.012	1.552	1.285
Phenylalanine	2.518	3.152	2.902	2.738	2.593
Threonine	2.144	5.958	3.144	3.762	2.562
Valine	2.118	4.019	3.197	3.101	2.631
**Non-essential AAs (NEAAs)**					
Cystine	–	–	–	–	–
GLU	14.151	17.095	14.544	15.802	14.332
Tyrosine	2.633	1.791	1.822	2.081	1.915
Glycine	9.645	2.508	1.588	6.252	5.191
Proline	3.187	5.635	13.618	3.590	5.982
Serine	3.387	4.556	3.373	3.971	3.382
Arginine	6.849	3.386	2.868	5.063	4.569
ASP	7.060	10.037	5.479	8.644	6.166
Alanine	2.612	5.629	2.216	3.942	2.277
∑ EAAs	23.354	39.321	29.251	29.307	24.824
∑ NEAAs	49.524	50.637	45.508	49.345	43.814
EAAs/NEAAs	0.472	0.777	0.643	0.594	0.567
∑ AAs	72.878	89.958	74.759	78.652	68.638

DS, High protein-energy ball formulated with Sukkari date paste and Samh seed's powder; DW, High protein-energy ball formulated with Sukkari date paste and WPC; DC, High protein-energy ball formulated with Sukkari date paste and CMP; DSW, High protein-energy ball formulated with Sukkari date paste, Samh seed's powder, and WPC; and DSC, High protein-energy ball formulated with Sukkari date paste, Samh seed's powder and CMP.

### 3.3 Fatty acids (FAs) profile

Although fat and fatty acid residues are substantial nutritive compounds, the kind and quantity of fat consumed in the case of illness protection should not be ignored. Table 5 shows different high-protein energy balls' relative fatty acids (FAs) distribution. The highest content of saturated fatty acids (SFA) was recorded in the balls composed of date and WPC, and this was attributed to the presence of WPC, which contained 6.25% fat, while fat component was absent in date fruit. Also, these balls were rich in caprylic, capric, lauric, palmitic, and stearic acids. Vaghela and Kllara (41) reported that the commercial WPC comprised a high content of all FAs except oleic and butyric acids. On the other hand, adding Samh powder to date increased the content of unsaturated fatty acids (USFA) in balls due to the greatest level of oleic and linoleic acids in Samh powder. Ahmed et al. (42) found that the Samh seeds flour was rich in USFA, particularly linoleic acid.

**Table 5:** Relative Fatty acids distribution (Area %) of different high-protein energy ball formulations.

**Fatty acid**	**DS**	**DW**	**DC**	**DSW**	**DSC**
**Saturated fatty acids**					
Caproic acid (C6:0)	–	0.49	0.13	0.53	0.13
Caprylic acid (C8:0)	0.01	0.61	0.09	0.07	0.07
Capric acid (C10:0)	0.01	1.12	0.12	0.17	0.08
Lauric acid (C12:0)	0.01	1.8	0.74	0.26	0.55
Tridecanoic acid (C13:0)	–	–	0.08	–	0.06
Myristic acid (C14:0)	0.25	7.01	11.11	1.17	8.63
Pentadecanoic acid (C15:0)	0.2	0.86	1.43	0.29	1.16
Palmitic acid (C16:0)	15.29	43.19	33.36	18.7	29.09
Margaric acid (C17:0)	0.37	0.48	0.98	0.4	0.84
Stearic acid (C18:0)	3.36	19.74	15.59	5.22	12.96
Arachidic acid (C20:0)	0.78	0.32	0.44	0.75	0.53
Heneicosylic acid (C21:0)	0.06	–	0.04	0.01	0.05
Behenic acid (C22)	0.38	0.13	0.1	0.35	0.16
Tricosylic acid (C23:0)	0.13	–	0.02	0.11	0.05
Lignoceric acid (C24)	0.27	0.13	0.03	0.24	0.08
**Un-saturated fatty acids**					
Myristoleic acid (C14:1)	–	0.38	0.76	0.05	0.58
Palmitoleic acid (C16:1)	0.3	0.85	8.53	0.36	6.69
Oleic acid (C18:1n-9c)	27.57	16.28	21.2	27.07	23.11
Linolelaidic acid (C18:2n6t)	0.91	-	0.22	0.91	0.44
Linoleic acid (LA: cis-9,10, C18:2)	48.24	4.28	3.42	41.66	13.09
α-Linolenic acid (ALA: cis-,12,15, C18:3n3)	1.13	0.55	0.75	1.02	0.83
γ-Linolenic acid (C18:3n6)	–	–	0.07	–	0.06
Eicosenoic acid (cis-11, C20:1)	0.44	–	0.25	0.38	0.29
Eicosadienoic acid (cis-11,14, C20:2n6)	0.23	0.98	0.09	0.16	0.12
Erucic acid (C22:1n9c)	–	–	0.06	–	0.05
Docosadienoic acid (C22:2n6)	0.01	–	–	–	–
Nervonic acid (C24:1n9)	–	–	0.02	–	0.01
Eicosatetraenoic acid (EPA: C20:5n3)	–	–	0.03	–	0.04
Docosahexaenoic acid (DHA: C22:6n3)	–	–	0.03	–	0.02
∑ SFAs	21.12	75.88	64.26	28.27	54.44
∑ USFAs	78.83	23.32	35.43	71.61	45.33
∑ FAs	99.95	99.2	99.69	99.88	99.77

DS, High protein-energy ball formulated with Sukkari date paste and Samh seed's powder; DW, High protein-energy ball formulated with Sukkari date paste and WPC; DC, High protein-energy ball formulated with Sukkari date paste and CMP; DSW, High protein-energy ball formulated with Sukkari date paste, Samh seed's powder and WPC; and DSC, High protein-energy ball formulated with Sukkari date paste, Samh seed's powder and CMP.

Furthermore, Al-Jassir et al. (43) observed that linoleic was the predominant fatty acid in Samh seeds, pursed by oleic and palmitic acids, which agrees with our findings. It is worth mentioning that all ball formulations did not demonstrate any of the trans-fatty acids that should be decreased from their consumption due to their negative effects on human health. Their absence is attributed to the loss of industrially hydrogenated vegetable oils in the ball formulations.

### 3.4 Phenolic and flavonoid compounds

The differences in the phenolic and flavonoids identified in high-protein energy ball formulations are presented in Table 6. Analysis of HPLC illustrated the presence of nine phenolic and seven flavonoid compounds at various levels in different ball formulations. Our data revealed that blending date paste with Samh powder has a significant effect on the phenolic and flavonoids compounds of ball formulations, especially p-coumaric, o-coumaric, ferulic, chlorogenic, rutin, kaempferol, myricetin, and hesperidin components. However, balls composed of date paste and WPC exhibited the highest amount of p-hydroxybenzoic, catechin, and catechol components because of the phenolic and flavonoid components of Sukkari date. In this context, Saleh et al. (25) reported that the Sukkari date contained a high content of rutin (8.10 mg/kg) and catechin (7.50 mg/kg). Alahyane et al. (44) declared that date fruits contained plenty of polyphenols, isoflavones, flavonoids, carotenoids, and phenolic acids including ferulic, caffeic, gallic, syringic, and coumaric acids. Ouamnina et al. (45) found that gallic acid was one of the prevalent phenolic compounds in date fruit. Concerning Samh seeds powder, Ahmed et al. (42) confirmed that gallic acid was the large phenolics in Samh seeds, followed by catechol, and catechin, while resveratrol was the lowest phenolics. Epidemiological studies have illustrated that consuming meals or food products enriched with phenolics could be valuable in maintaining human health or preventing difference disorders or diseases including cancer, atherosclerosis, diabetes, asthma, and cardiovascular diseases. In addition, phenolics are vastly applied in various industries for an assorted range of purposes. For instance, gallic acid acts as a food additive where its ester derivatives are combined into processed food products and a matrix of food packaging substances to maintain food products from oxidation and deterioration (46). Furthermore, it is applied as a raw substance in the industry of paints, color enhancers, inks, and pharmaceutical drugs (47–50).

**Table 6:** Phenolic and flavonoid compounds (mg/100 g) of different high-protein energy ball formulations.

**Compound**	**DS**	**DW**	**DC**	**DSW**	**DSC**
**Phenolic compounds**					
Gallic acid	0.043	–	–	0.078	0.042
Caffeic acid	0.092	0.049	0.033	0.047	0.051
Syringic acid	0.023	0.013	0.015	0.012	0.008
P-coumaric acid	0.535	0.019	0.041	0.211	0.296
Ferulic acid	0.260	0.030	0.038	0.048	0.059
o-coumaric acid	0.049	0.020	0.017	0.012	0.015
p-hydroxybenzoic acid	0.098	0.178	0.013	0.012	0.195
Chlorogenic acid	0.278	0.207	0.099	0.044	0.058
Vanillic acid	0.064	0.071	0.070	0.051	0.074
**Flavonoid compounds**					
Rutin	0.142	0.070	0.050	0.013	0.010
Quercetin	–	–	0.014	–	0.063
Apigenin	0.002	0.001	0.004	0.001	0.001
Kaempferol	0.008	–	–	–	–
Catechin	0.109	0.241	0.083	0.115	0.117
Myricetin	0.032	0.009	0.014	0.008	–
Catechol	0.027	0.039	–	0.030	0.017
Hesperidin	0.022	–	–	–	0.007

DS, High protein-energy ball formulated with Sukkari date paste and Samh seed's powder; DW, High protein-energy ball formulated with Sukkari date paste and WPC; DC, High protein-energy ball formulated with Sukkari date paste and CMP; DSW, High protein-energy ball formulated with Sukkari date paste, Samh seed's powder, and WPC; and DSC, High protein-energy ball formulated with Sukkari date paste, Samh seed's powder and CMP.

### 3.5 Antioxidant properties

The antioxidant capacity of various high-protein energy ball formulations was evaluated *in vitro* using two assay approaches including DPPH radical scavenging ability and reducing power (Table 7). A DPPH radical scavenging assay is a level-dependent technique; as the level of overall antioxidant compounds increases, more DPPH radical scavenging ability will occur, and vice versa. Regarding reducing power, it is a technique that depends on reducing the ferric ions/ferricyanide complicated to the Fe2+ form in the existence of antioxidant components in the antioxidant matrix. The complicated generated with blue color can be estimated at 700 nm. The results in this study indicated that the DPPH-radical scavenging ability ranged from 52.98% to 68.33% for all ball formulations without significant differences, except date-camel milk powder balls that exhibited the lowest ability. The need for digestion of bioactive substances found in camel milk proteins, including casein and whey, may stem from their requirement to be broken down in the gastrointestinal tract, as has been noted earlier (13) could be the reason. Contrariwise, somewhat significant variations among ball formulations in the reducing power values were observed. Date-Samh powder ball formulation and balls formulated of date, Samh powder and WPC showed the significantly highest reducing power value. Comprehensively, the antioxidant properties of the high-protein energy ball formulations pretend to be promptly relative to the used ingredients composition. In detail, the antioxidant capacity of Sukkari date paste and Samh powder is closely associated with the high content of phenolic and flavonoid compounds. Alfaris et al. (51) stated that various components contributed to the antioxidant properties of date including antioxidant enzymes, and considerable amounts of vitamins and minerals that can scavenge reactive oxygen species or free radicals. Hossain and Rahman (52) mentioned that the antioxidant ability of phenolics is essentially attributed to redox characteristics that make them perform as hydrogen granter agents, reducing compounds and singlet O_2_ deactivators. The existence of hydroxyl groups in the phenolic compounds increases their activity (53).

**Table 7:** Antioxidants properties of different high protein-energy balls formulation.

**High-protein energy balls**	**DPPH radical scavenging activity (μg ascorbic acid equivalent/g sample)**	**Reducing power (μg ascorbic acid equivalent/g sample)**
DS	63.72± 1.85^ab^	346.75± 64.67^a^
DW	68.33± 5.13^a^	325.86 ± 47.84^b^
DC	52.98± 3.46^b^	278.78± 68.67^c^
DSW	63.78 ± 2.43^ab^	328.494± 58.86^ab^
DSC	59.87 ± 2.61^ab^	294.07± 55.97^c^

DS, High protein-energy ball formulated with Sukkari date paste and Samh seed's powder; DW, High protein-energy ball formulated with Sukkari date paste and WPC; DC, High protein-energy ball formulated with Sukkari date paste and CMP; DSW, High protein-energy ball formulated with Sukkari date paste, Samh seed's powder, and WPC; and DSC, High protein-energy ball formulated with Sukkari date paste, Samh seed's powder, and CMP. Data in the same column with various superscript letters differ significantly.

Furthermore, flavonoid compounds exhibit antioxidant properties, but this capacity differs from one compound to another based on the position and number of OH groups in the B ring. Principally, an O-dihydroxylated structure of the B ring leads to great antioxidant ability due to this structure give more constancy on the aroxyl radical through the delocalization of electrons or acts as a favorable binding position for transition metal ions (45). Saleh et al. (25) declared that Sukkari dates have reducing power with 50%, and a positive correlation between total phenolic compounds and the reduction of lipid oxidation was observed. Zhang et al. (54) found that methanolic extracts of date fruits showed moderate antioxidant activity using MTT assay. Also, they observed that methanolic and water extracts of dates restrained lipid peroxidation at 58–82% and 50–67%, respectively. Concerning whey protein concentrate, the antioxidant ability may be due to the chelating of metal ions via lactoferrin and serum albumin. Furthermore, peptides or AAs of whey proteins can act as electron granter agents and might transform free radicals to more constant and may restrain lipid peroxidation through the capability of non-phosphorylated groups to chelate transition metals (55). In the last decades, whey protein concentrate or isolate products have been applied to boost the nutritional and health values of food products. Mann et al. (56) stated that supplementation of flavored milk beverages with WPC enhanced the antioxidant activity. Zeng et al. (57) reviewed the addition of whey protein products to fermented foods as having improved antioxidant activity.

### 3.6 *In vitro* digestability and degree of proteolysis

The content of free NH3 groups measured by OPA assay and proteolysis degree were used to express about the intro digestibility of high-protein energy ball formulations (Table 8). Incorporating WPC in ball formulations significantly enhanced the digestibility and proteolysis degree compared to other balls free from WPC. Balls composed of date paste and camel milk powder ranked second in the digestibility and proteolysis degree. Desai et al. (58) stated that the incorporation of protein sources (WPC or camel milk powder) generated more formed bonds between carbohydrates and proteins in ball formulations, while it may obstruct the phenolics bind leading to an increment in protein digestibility. Tang et al. (59) studied the effect of equal nitrogenous component levels of casein, soy, and whey proteins on the prompting of muscle protein biosynthesis and found that the whey protein ingestion enhanced a larger increase in essential and branched-chain AAs levels in the blood than that of other protein sources. Hall et al. (60) attributed that to the rapid digestion of whey proteins. Hulmi et al. (61) and Phillips (62) confirmed that high-quality protein food products compose great concentrations of branched-chain AAs, particularly Leu acid, and these AAs are crucial promoting agents for protein biosynthesis in the human body.

**Table 8:** *In vitro* digestibility of different high-protein energy balls formulations.

**High-protein energy balls**	**Free amino groups (**μ**mol L-leucin equivalent/ml extract)**		
	**Before digestion**	**After digestion**	**Degree of proteolysis**
DS	884.24 ± 117.83^a^	1,087.11 ± 80.87^b^	202.88 ± 67.20^c^
DW	779.52 ± 18.29^a^	1,704.89 ± 39.91^a^	925.37 ± 21.62^a^
DC	512.65 ± 53.61^b^	1,122.71 ± 116.96^b^	610.06 ± 63.35^b^
DSW	769.34 ± 78.36^a^	1,682.68 ± 170.95^a^	913.34 ± 92.59^a^
DSC	461.75 ± 136.87^b^	1,011.67 ± 298.58^b^	550.92 ± 16.00^d^

DS, High protein-energy ball formulated with Sukkari date paste and Samh seed's powder; DW, High protein-energy ball formulated with Sukkari date paste and WPC; DC, High protein-energy ball formulated with Sukkari date paste and CMP; DSW, High protein-energy ball formulated with Sukkari date paste, Samh seed's powder, and WPC; and DSC, High protein-energy ball formulated with Sukkari date paste, Samh seed's powder, and CMP. Data in the same column with various superscript letters differ significantly.

Regarding camel milk, α-lactalbumin exists in high concentration and has great similarity with α-lactalbumin in human milk. Salami et al. (63) declared that α-lactalbumin of camel milk showed a greater hydrolysis degree by trypsin and chymotrypsin than that of cow milk proteins, and they proposed that α-lactalbumin of camel milk is the best substrate for proteolytic enzymes in the intestine than cow milk proteins. Zou et al. (64) and Han et al. (65) mentioned that camel milk is rich in whey acidic protein and peptidoglycan recognition protein-1 compared to cow and human milk, and these proteins display the potential to confer health benefits when digested by infants. On the other hand, Butts et al. (66) reported that vegetable proteins contain nutritional agents or factors that form a more complicated protein network, leading to a decrease in the digestibility of protein. Regarding the dairy source of proteins, the lower degree of proteolysis appearing in samples containing CMP than in samples containing WPC may be due to the presence of fat, casein and whey proteins in CMP, which may interfere during *in vitro* digestibility (67). This finding is supported by Khalesi et al. (68), who reported that casein is a slow-digesting milk protein, while whey protein (WP) is a fast-digesting milk protein. They also reported that heat denaturation of whey protein boosts its digestibility by making cleavage sites more accessible to pepsin, but interactions with caseins in milk protein concentrate can reduce overall protein digestibility.

### 3.7 *In vitro* glycemic indices

The *in vitro* method is frequently employed as an initial assessment prior to undertaking more extensive studies on glycemic responses in human subjects. The glycemic index (GI) serves as a valuable indicator of the relative glycemic response of various foods and reflects the quality of their carbohydrate content. Glycemic load (GL) is a critical measure that evaluates the total carbohydrate impact on glucose response, incorporating both glycemic index (GI) and the overall carbohydrate content of a food item. While GI serves as an essential indicator of carbohydrate quality, it does not account for variations in individual serving sizes. Therefore, understanding GL is essential for accurately assessing the glycemic effect associated with specific portions of food. Research categorizes foods as follows: those with a GL of 10 or lower are classified as low GL, those with a GL between 11 and 20 as medium GL, and those with a GL of 20 or higher as high GL (69, 70). In this study, both the *in vitro* GI and the calculated glycemic load (GL) for a 25 g serving of prepared high-protein energy balls were evaluated. The results of this assessment are presented in Table 9. It clearly observed that all high-protein energy ball formulations demonstrated low GI and GL levels. However, it is important to note that significant variations were observed among the different samples. Although the per cent of total carbohydrates (Table 2) was about 71% and 69.7% in DSW and DS samples, respectively, the highest GI was for the sample formulated with date paste, Samh seeds powder and WPC (DSW: 40.219 ± 0.033), while the least was for the sample only formulated with date paste and Samh seeds powder (DS: 40.053 ± 0.019). This may be due to the presence of lactose in the WPC, which releases more glucose during *in vitro* digestion. Regarding the rest of the samples (DW, DC and DSC), no highly significant differences were detected in GI. As previously reported by Sulung et al. (69), several factors influence the GI of food beyond just the carbohydrate content. These factors include particle size, preparation methods, cooking techniques, food processing, the physical form of the food, and the presence of different macronutrients such as proteins and fats. Notably, having a higher proportion of protein or fat in a carbohydrate-rich meal can help mitigate the glycemic response by slowing gastric emptying and enhancing insulin secretion. In contrast, significant differences (*p* < 0.05) in glycemic load (GL) were observed among all samples tested. The highest GL was found in the DS sample (7.118 ± 0.004), while the lowest was in the DC sample (6.430 ± 0.003), followed by the DSC sample (6.547 ± 0.021). Additionally, the inclusion of CM powder in the formula resulted in a lower GL compared to other samples that did not contain CM powder. Previously, Mohammadabadi et al. (71) reported that the insulin-like proteins and small immunoglobulins in camel milk aid in regenerating β-cells and regulating blood glucose levels. Furthermore, these substances replicate insulin's effects and improve glucose absorption, leading to a reduction in blood glucose levels (72). The elevated GL observed in the formulation containing date paste and Samh seeds can be attributed to its principal carbohydrates: glucose, sucrose, maltose, and fructose. Notably, glucose, sucrose, and maltose exhibit higher GI values compared to lactose (70).

**Table 9:** *In vitro* GI and GL of different high protein-energy balls formulation.

**High-protein energy balls**	**GI**	GL^*^
DS	40.053 ± 0.019^c^	7.118 ± 0.004^a^
DW	40.062 ± 0.005^bc^	6.842 ± 0.004^c^
DC	40.117 ± 0.009^b^	6.430 ± 0.003^e^
DSW	40.219 ± 0.033^a^	6.980 ± 0.066^b^
DSC	40.092 ± 0.016^bc^	6.547 ± 0.021^d^

^*^The GL was calculated for a single recommended serving size of 25 g for each sample. DS, High protein-energy ball formulated with Sukkari date paste and Samh seed's powder; DW, High protein-energy ball formulated with Sukkari date paste and WPC; DC, High protein-energy ball formulated with Sukkari date paste and CMP; DSW, High protein-energy ball formulated with Sukkari date paste, Samh seed's powder and WPC; and DSC, High protein-energy ball formulated with Sukkari date paste, Samh seed's powder and CMP. Data in the same column with various superscript letters differ significantly.

### 3.8 Sensory assessment of different high-protein energy balls formulation

The association of multifunctional compounds or ingredients to prepare innovative food may change the sensory attributes of the obtained product, leading to decreased consumer acceptability. Hence, it is substantial to investigate the alterations in sensory characteristics of high-protein energy ball formulations as a result of utilizing date paste, Samh seeds powder, and different origins of milk proteins. The results of sensory assessment (Table 10) showed that all high-protein energy ball formulations were acceptable by panelists. The hedonic scale test demonstrated that the appearance, texture, and overall acceptability are recognized without significant variations among high-protein ball formulations except date paste-WPC ball (DW) formulation. Panelists gained a significant lower score for flavor of balls formulated of date paste and Samh powder or date paste and WPC compared to other ball formulations. Our findings indicated that balls formulated of date paste and WPC exhibited a soft and sticky texture, while adding Samh seeds powder to ball formulations contributed to a harder texture. Hogan et al. (73) and Szydłowska et al. (1) reported that the utilization of WPC in high-protein formulation bars maintained a softer texture of bars. Ibrahim et al. (74) found that application of date paste gave a sweet flavor to snake bars and formulation bars containing 50% date paste exhibited the highest overall acceptability and sensory attributes.

**Table 10:** Sensory scores of different high-protein energy balls formulation.

**High-protein energy balls**	**Appearance**	**Flavor**	**Aroma**	**Texture**	**Overall**
DS	6.77 ± 1.79^ab^	6.46 ± 2.11^b^	6.92 ± 1.89^a^	6.62 ± 1.19^ab^	6.85 ± 1.68^ab^
DW	6.69 ± 2.39^b^	6.54 ± 1.90^b^	6.15 ± 2.73^a^	5.62 ± 1.80^b^	6.15 ± 1.77^b^
DC	8.08 ± 1.04^a^	7.85 ± 1.34^a^	7.23 ± 1.83^a^	7.46 ± 1.56^a^	7.62 ± 1.39^a^
DSW	7.54 ± 1.13^ab^	7.15 ± 0.80^ab^	7.62 ± 0.96^a^	7.38 ± 1.12^a^	7.15 ± 0.80^ab^
DSC	7.08 ± 2.06^ab^	7.00 ± 1.68^ab^	6.92 ± 1.89^a^	6.85 ± 1.68^a^	7.00 ± 1.87^ab^

DS, High protein-energy ball formulated with Sukkari date paste and Samh seed's powder; DW, High protein-energy ball formulated with Sukkari date paste and WPC; DC, High protein-energy ball formulated with Sukkari date paste and CMP; DSW, High protein-energy ball formulated with Sukkari date paste, Samh seed's powder and WPC; and DSC High protein-energy ball formulated with Sukkari date paste, Samh seed's powder, and CMP. Data in the same column with various superscript letters differ significantly.

Panelists declared that more than 90% of consumers particularly athletes would purchase such balls if they available on the market. Several research works emphasize that a gap found desirable behaviors and interests on the one hand and the realistic purchase power of consumers on the other hand. Research works realize that favorable consumer buying attitude to healthy high-protein balls exists essentially among the richest consumers. Other obstacles include health and price awareness, sensitivity, the comparison between value and price, and the desire to pay. Pricing of healthy or functional food products, particularly sports products, is a multifaceted and contradictory concern because consumers desirable to buy products with low prices with high quality at the same time (75, 76). Therefore, scientific research seeks constantly to minimize the gap between consumer desires and the prices of healthy or functional products.

### 3.9 Glycemic response in healthy young adults

The term Glycemic response refers to the changes in blood glucose levels following the consumption of carbohydrates containing meals. The high levels of blood glucose after meals are associated with diet-related diseases, such as obesity, type 2 diabetes, and cardiovascular problems (70, 77).

**Figure 3:**
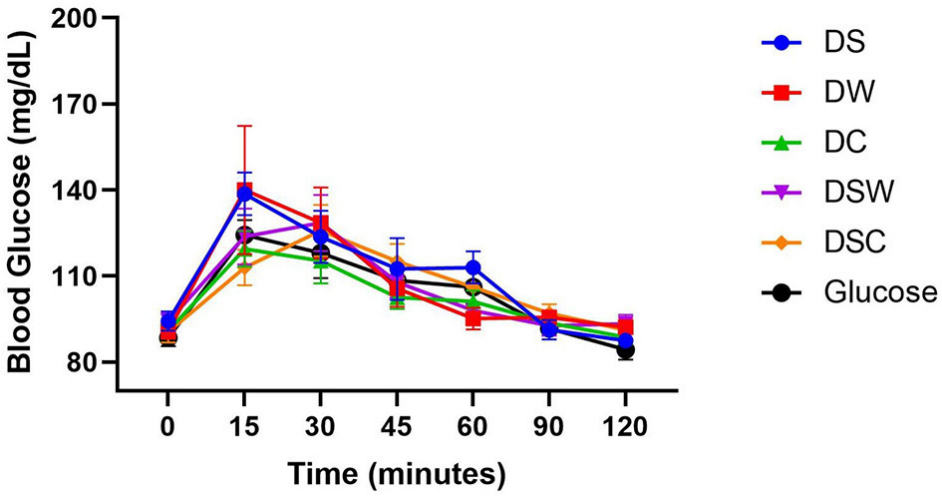
Glycemic response of different high protein-energy balls in healthy young adults. DS, High protein-energy ball formulated with Sukkari date paste and Samh seed's powder; DW, High protein-energy ball formulated with Sukkari date paste and WPC; DC, High protein-energy ball formulated with Sukkari date paste and CMP; DSW, High protein-energy ball formulated with Sukkari date paste, Samh seed's powder and WPC; and DSC, High protein-energy ball formulated with Sukkari date paste, Samh seed's powder and CMP.

The data illustrating the average fasting blood glucose levels, measured at baseline (0 min), alongside the blood glucose responses in healthy young adults following the consumption of a standard 50 g glucose solution and various high-protein energy balls, is presented in Figure 3. Blood glucose measurements were taken at 15, 30, 45, 60, 90, and 120 min post-ingestion (34). The results indicate the average fasting blood glucose level among the participants was 88.5 ± 7.15 mg/dL, which falls within the normal range. The postprandial blood glucose response exhibited a consistent pattern across most samples. Blood glucose levels increased from baseline, often peaking at 15 min, and returned to baseline by 120 min, except for the DSC and DSW samples. The highest blood glucose levels for both DSC and DSW were observed 30 min after ingestion. The slow gastric emptying that follows the consumption of DSC and DSW samples may explain why there is a delayed increase in blood glucose levels during the feeding state. This may occur because delayed emptying allows glucose to enter circulation gradually. This phenomenon was previously noted by Shkembi and Huppertz (70). At minute 15, the highest blood glucose levels were 140 ± 54.80 mg/dl and 139 ± 18.12917 mg/dl for DW and DS samples, respectively, while the lowest was for DSC (113± 15.14 mg/dl) and DC (120± 15.23 mg/dl) samples. The observed highest glycemic responses for the DW and DS samples may not be solely attributable to their sugar content but rather to their overall composition. As indicated by Dhaheri et al. (78), the GI is highly influenced by the levels of fructose and sucrose present. These sugars are predominant in dates, as highlighted by Alzahrani et al. (79). Consequently, it can be inferred that the higher proportion of date paste in the DW samples (90%) compared to the DS samples (59%) may be a contributing factor to their increased glycemic response concerning the other samples. All samples represented a gradual decline in blood glucose levels after 30 min, with a return to pre-meal levels observed by the 120-min mark. This data suggests that there was no occurrence of post-meal hyperglycemia. Depending on these findings, the high-protein energy balls formulated with Samh seeds powder and dairy proteins, especially from camel milk, could be recommended as a source of protein and energy food between meals for healthy young adults.

## Data Availability

The original contributions presented in the study are included in the article/supplementary material, further inquiries can be directed to the corresponding author.
